# 3-Isobutyl-5,5-di­phenyl­imidazolidine-2,4-dione

**DOI:** 10.1107/S2414314622005983

**Published:** 2022-06-10

**Authors:** Walid Guerrab, Abderrazzak El Moutaouakil Ala Allah, Abdulsalam Alsubari, Joel T. Mague, Youssef Ramli

**Affiliations:** a Laboratory of Medicinal Chemistry, Drug Sciences Research Center, Faculty of Medicine and Pharmacy, Mohammed V University in Rabat, Morocco; b Laboratory of Medicinal Chemistry, Faculty of Clinical Pharmacy, 21 September University, Yemen; c Department of Chemistry, Tulane University, New Orleans, LA 70118, USA; Sunway University, Malaysia

**Keywords:** crystal structure, imidazolidinedione, hydrogen bond, C—H⋯π(ring) inter­action

## Abstract

The imidazolidine ring in the title mol­ecule, C_19_H_20_N_2_O_2_, is slightly ‘ruffled’. In the crystal, a layer structure is generated by N—H⋯O and C—H⋯O hydrogen bonds plus C—H⋯π(ring) inter­actions.

## Abstract 2

The imidazolidine ring is slightly ‘ruffled’ and the isobutyl substituent is rotated well out of the plane of its ring. In the crystal, inversion dimers are formed by pairs of N—H⋯O hydrogen bonds with C—H⋯O hydrogen bonds linking them into chains parallel to (10 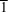 ). The chains are joined into layers parallel to the *ac* plane by C—H⋯π(ring) inter­actions.

## Graphical abstract

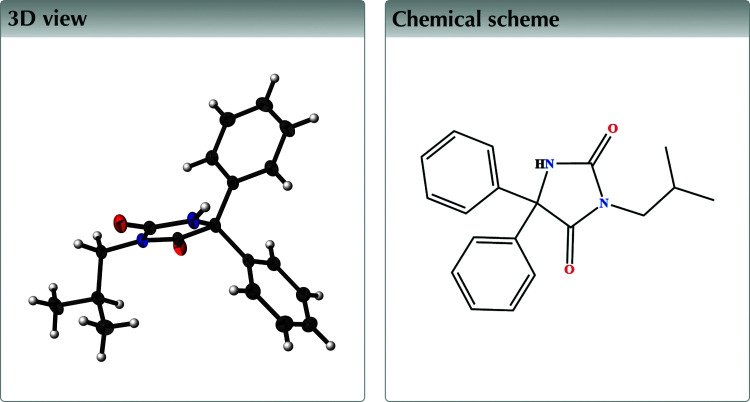

## Structure description

Imidazolidin-2,4-dione, also known as hydantoin, is an important nucleus found in numerous natural products and in several clinically important medicines. One of the best known examples of such a derivative is phenytoine, 5,5-di­phenyl­imidazolidine-2,4-dione, a drug widely prescribed as an anti­convulsant agent and for the treatment of many other diseases including HIV (Weichet, 1974 ▸; Havera & Strycker, 1976 ▸; Khodair *et al.*, 1997 ▸; Thenmozhiyal *et al.*, 2004 ▸).

**Figure 1:**
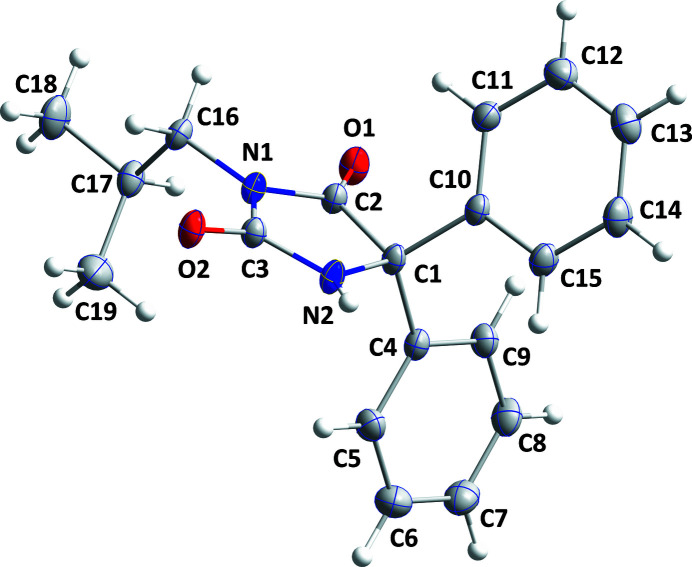
The title mol­ecule with the labelling scheme and 50% probability ellipsoids.

Given the wide range of therapeutic applications for such compounds, and in a continuation of our work in this area (Ramli *et al.*, 2017*a* ▸,*b* ▸; Akrad *et al.* 2017 ▸; Guerrab *et al.* 2019 ▸, 2020*a* ▸,*b* ▸, 2021 ▸, 2022 ▸), the title compound (Fig. 1 ▸) was prepared and its crystal structure is reported here.

**Figure 2:**
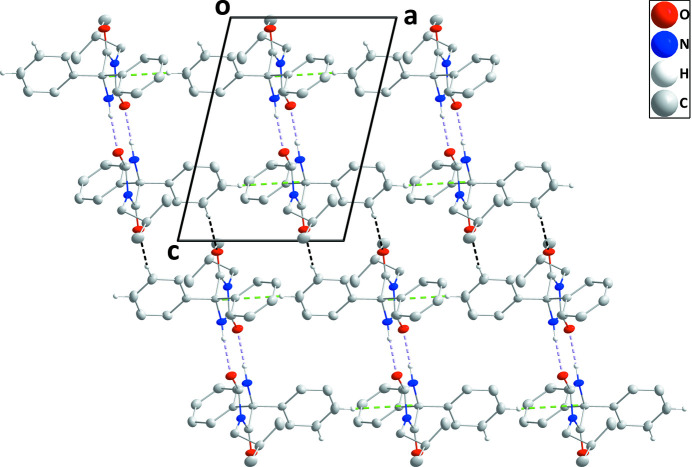
A portion of one layer viewed along the *b*-axis direction with N—H⋯O and C—H⋯O hydrogen bonds depicted, respectively, by violet and black dashed lines. C—H⋯π(ring) inter­actions are depicted by green dashed lines and non-inter­acting hydrogen atoms are omitted for clarity.

**Figure 3:**
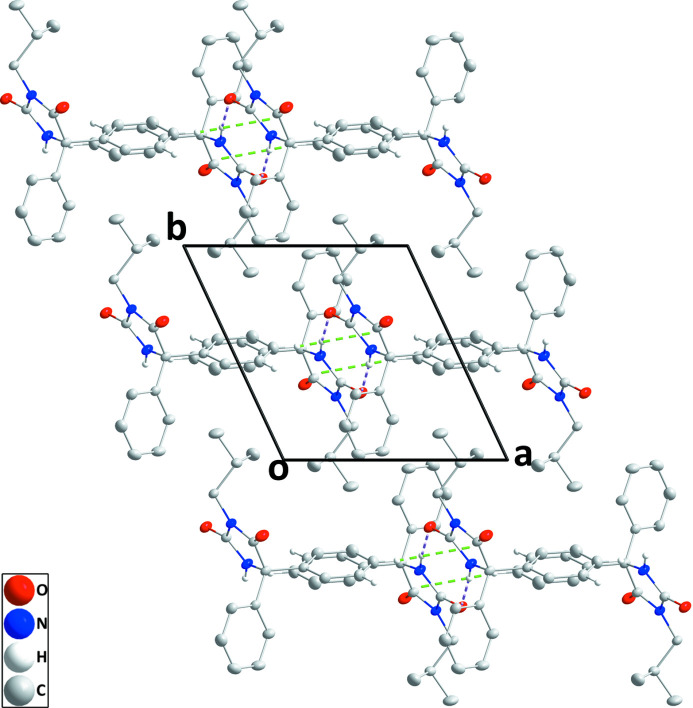
Packing viewed along the *c*-axis direction with inter­molecular inter­actions depicted as in Fig. 2 ▸ and non-inter­acting hydrogen atoms omitted for clarity.

The two phenyl rings (C4–C9 and C10–C15) are disposed on either side of the five-membered ring and make dihedral angles of 68.42 (3) and 73.04 (3)°, respectively, with the mean plane of the latter ring. The five-membered ring is slightly ‘ruffled’ with deviations from the mean plane ranging from 0.206 (5) Å (N2) to −0.218 (5) Å (C3) (r.m.s. deviation = 0.0155 Å). The isobutyl group is rotated well out of the mean plane of the five-membered ring, as indicated by the C2—N1—C16—C17 torsion angle of 72.64 (10)°. In the crystal, inversion dimers are formed by pairs of N2—H2⋯O2 hydrogen bonds (Table 1 ▸) with the dimers connected by C8—H8⋯O1 hydrogen bonds, forming chains of mol­ecules extending parallel to (10 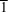 ) (Fig. 2 ▸ and Table 2 ▸). The chains are connected into layers parallel to the *ac* plane by C7—H7⋯*Cg*1 inter­actions (Table 1 ▸ and Fig. 3 ▸).

## Synthesis and crystallization

To a solution of 5,5-di­phenyl­imidazolidine-2,4-dione (500 mg, 1.98 mmol), one equivalent of isobutyl bromide (246.88 mL, 1.98 mmol) in absolute di­methyl­formamide (DMF, 15 ml) was added and the resulting solution heated under reflux for 3 h in the presence of 1.1 equivalents of K_2_CO_3_ (301.31 mg, 2.18 mmol). The reaction mixture was filtered while hot, and the solvent evaporated under reduced pressure. The residue obtained was dried and recrystallized from an ethanol solution to yield colourless prism-like crystals (Guerrab *et al.*, 2018 ▸)

## Refinement

Crystal data, data collection and structure refinement details are presented in Table 2 ▸. A small amount of residual density, well removed from the main mol­ecule and which could not be satisfactorily modelled by a plausible solvent mol­ecule disordered across a centre of symmetry was removed with *PLATON* SQUEEZE (Spek, 2015 ▸). Three reflections affected by the beamstop were omitted from the final refinement.

## Supplementary Material

Crystal structure: contains datablock(s) global, I. DOI: 10.1107/S2414314622005983/tk4078sup1.cif

Structure factors: contains datablock(s) I. DOI: 10.1107/S2414314622005983/tk4078Isup2.hkl

Supporting information file. DOI: 10.1107/S2414314622005983/tk4078Isup3.cml

CCDC reference: 2176804

Additional supporting information:  crystallographic information; 3D view; checkCIF report

## Figures and Tables

**Table 1:** Hydrogen-bond geometry (Å, °) *Cg*1 is the centroid of the five-membered ring.

*D*—H⋯*A*	*D*—H	H⋯*A*	*D*⋯*A*	*D*—H⋯*A*
N2—H2⋯O2^i^	0.91 (1)	1.95 (1)	2.8512 (9)	174 (1)
C7—H7⋯*Cg*1^ii^	0.95	2.99	3.9308 (13)	170
C8—H8⋯O1^iii^	0.95	2.46	3.4069 (13)	172

Symmetry codes: (i) 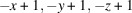 ; (ii) 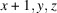 ; (iii) 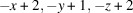 .

**Table 2:** Experimental details

Crystal data	
Chemical formula	C_19_H_20_N_2_O_2_
*M*_r_	308.37
Crystal system, space group	Triclinic, *P* 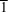
Temperature (K)	150
*a*, *b*, *c* (Å)	8.9747 (7), 9.7306 (7), 11.8780 (8)
α, β, γ (°)	104.676 (3), 96.334 (3), 112.243 (3)
*V* (Å^3^)	903.81 (12)
*Z*	2
Radiation type	Mo *K*α
μ (mm^−1^)	0.07
Crystal size (mm)	0.46 × 0.41 × 0.13

Data collection	
Diffractometer	Bruker D8 QUEST PHOTON 3 diffractometer
Absorption correction	Numerical (*SADABS*; Krause *et al.*, 2015 ▸)
*T*_min_, *T*_max_	0.93, 0.99
No. of measured, independent and observed [*I* > 2σ(*I*)] reflections	42215, 6214, 5222
*R*_int_	0.040
(sin θ/λ)_max_ (Å^−1^)	0.755

Refinement	
*R*[*F* ^2^ > 2σ(*F* ^2^)], *wR*(*F* ^2^), *S*	0.044, 0.128, 1.05
No. of reflections	6214
No. of parameters	213
No. of restraints	1
H-atom treatment	H atoms treated by a mixture of independent and constrained refinement
Δρ_max_, Δρ_min_ (e Å^−3^)	0.41, −0.19

Computer programs: *APEX4* and *SAINT* (Bruker, 2021 ▸), *SHELXT* (Sheldrick, 2015*a* ▸), *SHELXL2018/1* (Sheldrick, 2015*b* ▸), *DIAMOND* (Brandenburg & Putz, 2012 ▸) and *SHELXTL* (Sheldrick, 2008 ▸).

## full crystallographic data

### Crystal data

**Table:**

C_19_H_20_N_2_O_2_	*Z* = 2
*M**_r_* = 308.37	*F*(000) = 328
Triclinic, *P*1	*D*_x_ = 1.133 Mg m^−^^3^
*a* = 8.9747 (7) Å	Mo *K*α radiation, λ = 0.71073 Å
*b* = 9.7306 (7) Å	Cell parameters from 9909 reflections
*c* = 11.8780 (8) Å	θ = 2.5–31.9°
α = 104.676 (3)°	µ = 0.07 mm^−^^1^
β = 96.334 (3)°	*T* = 150 K
γ = 112.243 (3)°	Thick plate, colourless
*V* = 903.81 (12) Å^3^	0.46 × 0.41 × 0.13 mm

### Data collection

**Table:**

Bruker D8 QUEST PHOTON 3 diffractometer	6214 independent reflections
Radiation source: fine-focus sealed tube	5222 reflections with *I* > 2σ(*I*)
Graphite monochromator	*R*_int_ = 0.040
Detector resolution: 7.3910 pixels mm^-1^	θ_max_ = 32.5°, θ_min_ = 2.5°
φ and ω scans	*h* = −13→13
Absorption correction: numerical (*SADABS*; Krause *et al.*, 2015)	*k* = −14→14
*T*_min_ = 0.93, *T*_max_ = 0.99	*l* = −17→17
42215 measured reflections	

### Refinement

**Table:**

Refinement on *F*^2^	Primary atom site location: dual
Least-squares matrix: full	Secondary atom site location: difference Fourier map
*R*[*F*^2^ > 2σ(*F*^2^)] = 0.044	Hydrogen site location: mixed
*wR*(*F*^2^) = 0.128	H atoms treated by a mixture of independent and constrained refinement
*S* = 1.05	*w* = 1/[σ^2^(*F*_o_^2^) + (0.0689*P*)^2^ + 0.1685*P*] where *P* = (*F*_o_^2^ + 2*F*_c_^2^)/3
6214 reflections	(Δ/σ)_max_ = 0.001
213 parameters	Δρ_max_ = 0.41 e Å^−^^3^
1 restraint	Δρ_min_ = −0.19 e Å^−^^3^

### Special details

**Table:**

Experimental. The diffraction data were obtained from 9 sets of frames, each of width 0.5° in ω or φ, collected with scan parameters determined by the "strategy" routine in *APEX3*. The scan time was 5 sec/frame.
Geometry. All esds (except the esd in the dihedral angle between two l.s. planes) are estimated using the full covariance matrix. The cell esds are taken into account individually in the estimation of esds in distances, angles and torsion angles; correlations between esds in cell parameters are only used when they are defined by crystal symmetry. An approximate (isotropic) treatment of cell esds is used for estimating esds involving l.s. planes.
Refinement. Refinement of F^2^ against ALL reflections. The weighted R-factor wR and goodness of fit S are based on F^2^, conventional R-factors R are based on F, with F set to zero for negative F^2^. The threshold expression of F^2^ > 2sigma(F^2^) is used only for calculating R-factors(gt) etc. and is not relevant to the choice of reflections for refinement. R-factors based on F^2^ are statistically about twice as large as those based on F, and R- factors based on ALL data will be even larger. H-atoms attached to carbon were placed in calculated positions (C—H = 0.95 - 1.00 Å) and were included as riding contributions with isotropic displacement parameters 1.2 - 1.5 times those of the attached atoms. That attached to nitrogen was placed in a location derived from a difference map and refined with a DFIX 0.91 0.01 instruction. A small amount of residual density, well-removed from the main molecule and which could not be satisfactorily modeled by a plausible solvent molecule disordered across a center of symmetry was removed with *PLATON SQUEEZE* (Spek, 2015). Three reflections affected by the beamstop were omitted from the final refinement.

### Fractional atomic coordinates and isotropic or equivalent isotropic displacement parameters (Å^2^)

**Table:**

	*x*	*y*	*z*	*U*_iso_*/*U*_eq_	
O1	0.74629 (9)	0.64608 (8)	0.94977 (5)	0.02535 (14)	
O2	0.51349 (8)	0.68712 (7)	0.60449 (5)	0.02359 (14)	
N1	0.63385 (9)	0.70411 (8)	0.79418 (6)	0.01786 (13)	
N2	0.62040 (9)	0.51465 (8)	0.63706 (6)	0.01972 (14)	
H2	0.5822 (15)	0.4472 (13)	0.5613 (8)	0.030*	
C1	0.68961 (10)	0.48254 (9)	0.73985 (7)	0.01730 (14)	
C2	0.69602 (10)	0.61866 (9)	0.84376 (7)	0.01818 (15)	
C3	0.58232 (10)	0.63811 (9)	0.66914 (7)	0.01764 (15)	
C4	0.86580 (10)	0.49751 (9)	0.74187 (7)	0.01831 (15)	
C5	0.95614 (12)	0.56243 (11)	0.66554 (8)	0.02490 (17)	
H5	0.908433	0.598669	0.610614	0.030*	
C6	1.11659 (13)	0.57417 (12)	0.66981 (9)	0.0306 (2)	
H6	1.177780	0.617680	0.617333	0.037*	
C7	1.18692 (12)	0.52243 (12)	0.75054 (10)	0.0305 (2)	
H7	1.295578	0.529077	0.752362	0.037*	
C8	1.09899 (11)	0.46090 (11)	0.82877 (9)	0.02727 (18)	
H8	1.148247	0.427406	0.885034	0.033*	
C9	0.93872 (11)	0.44843 (10)	0.82459 (8)	0.02205 (16)	
H9	0.878635	0.406433	0.878110	0.026*	
C10	0.57204 (10)	0.32284 (9)	0.74399 (7)	0.01842 (15)	
C11	0.45908 (11)	0.30670 (10)	0.81674 (8)	0.02336 (17)	
H11	0.459215	0.397322	0.870843	0.028*	
C12	0.34570 (12)	0.15808 (12)	0.81053 (9)	0.02819 (19)	
H12	0.268623	0.147944	0.860092	0.034*	
C13	0.34519 (12)	0.02510 (11)	0.73224 (9)	0.02882 (19)	
H13	0.267373	−0.075954	0.727691	0.035*	
C14	0.45904 (13)	0.04021 (11)	0.66037 (9)	0.02807 (19)	
H14	0.459695	−0.050708	0.607264	0.034*	
C15	0.57189 (11)	0.18826 (10)	0.66618 (8)	0.02336 (17)	
H15	0.649371	0.197963	0.616936	0.028*	
C16	0.61826 (10)	0.84333 (9)	0.86303 (7)	0.01963 (15)	
H16A	0.564388	0.820207	0.928583	0.024*	
H16B	0.545921	0.868521	0.810459	0.024*	
C17	0.78453 (11)	0.98600 (10)	0.91629 (8)	0.02349 (17)	
H17	0.854577	0.961276	0.972365	0.028*	
C18	0.75511 (15)	1.12407 (11)	0.98774 (10)	0.0339 (2)	
H18A	0.689042	1.151726	0.933699	0.051*	
H18B	0.861657	1.214255	1.026831	0.051*	
H18C	0.695748	1.094763	1.048519	0.051*	
C19	0.87543 (13)	1.02639 (13)	0.81979 (11)	0.0353 (2)	
H19A	0.899177	0.938670	0.778900	0.053*	
H19B	0.979417	1.120428	0.856671	0.053*	
H19C	0.806095	1.046019	0.761788	0.053*	

### Atomic displacement parameters (Å^2^)

**Table:**

	*U*^11^	*U*^22^	*U*^33^	*U*^12^	*U*^13^	*U*^23^
O1	0.0371 (4)	0.0246 (3)	0.0156 (3)	0.0173 (3)	0.0006 (2)	0.0042 (2)
O2	0.0304 (3)	0.0223 (3)	0.0201 (3)	0.0158 (2)	−0.0010 (2)	0.0056 (2)
N1	0.0219 (3)	0.0160 (3)	0.0157 (3)	0.0104 (2)	0.0007 (2)	0.0029 (2)
N2	0.0279 (3)	0.0191 (3)	0.0142 (3)	0.0143 (3)	0.0004 (2)	0.0036 (2)
C1	0.0225 (3)	0.0167 (3)	0.0143 (3)	0.0110 (3)	0.0017 (3)	0.0046 (2)
C2	0.0218 (3)	0.0165 (3)	0.0170 (3)	0.0099 (3)	0.0027 (3)	0.0046 (3)
C3	0.0195 (3)	0.0165 (3)	0.0165 (3)	0.0083 (3)	0.0016 (3)	0.0045 (3)
C4	0.0213 (3)	0.0159 (3)	0.0178 (3)	0.0095 (3)	0.0019 (3)	0.0041 (3)
C5	0.0286 (4)	0.0262 (4)	0.0242 (4)	0.0135 (3)	0.0073 (3)	0.0115 (3)
C6	0.0283 (4)	0.0324 (5)	0.0332 (5)	0.0124 (4)	0.0119 (4)	0.0128 (4)
C7	0.0218 (4)	0.0295 (4)	0.0395 (5)	0.0114 (3)	0.0058 (4)	0.0097 (4)
C8	0.0235 (4)	0.0257 (4)	0.0329 (4)	0.0117 (3)	0.0001 (3)	0.0106 (3)
C9	0.0231 (4)	0.0214 (4)	0.0227 (4)	0.0102 (3)	0.0022 (3)	0.0087 (3)
C10	0.0213 (3)	0.0174 (3)	0.0176 (3)	0.0104 (3)	0.0015 (3)	0.0050 (3)
C11	0.0256 (4)	0.0228 (4)	0.0246 (4)	0.0133 (3)	0.0069 (3)	0.0071 (3)
C12	0.0261 (4)	0.0289 (4)	0.0311 (4)	0.0109 (3)	0.0088 (3)	0.0122 (4)
C13	0.0293 (4)	0.0214 (4)	0.0303 (4)	0.0058 (3)	0.0018 (3)	0.0094 (3)
C14	0.0344 (5)	0.0177 (4)	0.0270 (4)	0.0099 (3)	0.0018 (3)	0.0029 (3)
C15	0.0287 (4)	0.0190 (4)	0.0212 (4)	0.0112 (3)	0.0048 (3)	0.0030 (3)
C16	0.0211 (3)	0.0162 (3)	0.0212 (3)	0.0103 (3)	0.0024 (3)	0.0025 (3)
C17	0.0223 (4)	0.0167 (3)	0.0269 (4)	0.0078 (3)	−0.0009 (3)	0.0030 (3)
C18	0.0423 (5)	0.0190 (4)	0.0335 (5)	0.0128 (4)	0.0026 (4)	−0.0001 (3)
C19	0.0294 (5)	0.0288 (5)	0.0462 (6)	0.0090 (4)	0.0126 (4)	0.0132 (4)

### Geometric parameters (Å, º)

**Table:**

O1—C2	1.2139 (10)	C10—C15	1.3980 (11)
O2—C3	1.2259 (9)	C11—C12	1.3956 (13)
N1—C2	1.3698 (10)	C11—H11	0.9500
N1—C3	1.4045 (10)	C12—C13	1.3870 (14)
N1—C16	1.4598 (10)	C12—H12	0.9500
N2—C3	1.3465 (10)	C13—C14	1.3916 (15)
N2—C1	1.4652 (10)	C13—H13	0.9500
N2—H2	0.906 (8)	C14—C15	1.3913 (13)
C1—C4	1.5289 (11)	C14—H14	0.9500
C1—C10	1.5295 (11)	C15—H15	0.9500
C1—C2	1.5425 (11)	C16—C17	1.5273 (12)
C4—C5	1.3939 (12)	C16—H16A	0.9900
C4—C9	1.3979 (11)	C16—H16B	0.9900
C5—C6	1.3948 (13)	C17—C19	1.5250 (14)
C5—H5	0.9500	C17—C18	1.5282 (13)
C6—C7	1.3868 (14)	C17—H17	1.0000
C6—H6	0.9500	C18—H18A	0.9800
C7—C8	1.3895 (15)	C18—H18B	0.9800
C7—H7	0.9500	C18—H18C	0.9800
C8—C9	1.3911 (12)	C19—H19A	0.9800
C8—H8	0.9500	C19—H19B	0.9800
C9—H9	0.9500	C19—H19C	0.9800
C10—C11	1.3930 (12)		
			
C2—N1—C3	111.47 (6)	C10—C11—C12	120.34 (8)
C2—N1—C16	124.21 (7)	C10—C11—H11	119.8
C3—N1—C16	124.29 (6)	C12—C11—H11	119.8
C3—N2—C1	112.87 (6)	C13—C12—C11	120.22 (9)
C3—N2—H2	120.9 (8)	C13—C12—H12	119.9
C1—N2—H2	124.6 (8)	C11—C12—H12	119.9
N2—C1—C4	112.60 (7)	C12—C13—C14	119.79 (9)
N2—C1—C10	109.66 (6)	C12—C13—H13	120.1
C4—C1—C10	112.73 (6)	C14—C13—H13	120.1
N2—C1—C2	100.71 (6)	C15—C14—C13	120.09 (9)
C4—C1—C2	108.58 (6)	C15—C14—H14	120.0
C10—C1—C2	111.97 (6)	C13—C14—H14	120.0
O1—C2—N1	125.93 (7)	C14—C15—C10	120.44 (8)
O1—C2—C1	126.97 (7)	C14—C15—H15	119.8
N1—C2—C1	107.10 (6)	C10—C15—H15	119.8
O2—C3—N2	128.11 (7)	N1—C16—C17	112.91 (7)
O2—C3—N1	124.19 (7)	N1—C16—H16A	109.0
N2—C3—N1	107.69 (6)	C17—C16—H16A	109.0
C5—C4—C9	119.56 (8)	N1—C16—H16B	109.0
C5—C4—C1	121.55 (7)	C17—C16—H16B	109.0
C9—C4—C1	118.87 (7)	H16A—C16—H16B	107.8
C4—C5—C6	119.99 (8)	C19—C17—C16	111.65 (8)
C4—C5—H5	120.0	C19—C17—C18	111.13 (8)
C6—C5—H5	120.0	C16—C17—C18	108.81 (8)
C7—C6—C5	120.10 (9)	C19—C17—H17	108.4
C7—C6—H6	120.0	C16—C17—H17	108.4
C5—C6—H6	120.0	C18—C17—H17	108.4
C6—C7—C8	120.22 (9)	C17—C18—H18A	109.5
C6—C7—H7	119.9	C17—C18—H18B	109.5
C8—C7—H7	119.9	H18A—C18—H18B	109.5
C7—C8—C9	119.90 (8)	C17—C18—H18C	109.5
C7—C8—H8	120.1	H18A—C18—H18C	109.5
C9—C8—H8	120.1	H18B—C18—H18C	109.5
C8—C9—C4	120.21 (8)	C17—C19—H19A	109.5
C8—C9—H9	119.9	C17—C19—H19B	109.5
C4—C9—H9	119.9	H19A—C19—H19B	109.5
C11—C10—C15	119.12 (8)	C17—C19—H19C	109.5
C11—C10—C1	122.52 (7)	H19A—C19—H19C	109.5
C15—C10—C1	118.25 (7)	H19B—C19—H19C	109.5
			
C3—N2—C1—C4	−118.52 (8)	C1—C4—C5—C6	−179.97 (8)
C3—N2—C1—C10	115.10 (8)	C4—C5—C6—C7	−0.49 (15)
C3—N2—C1—C2	−3.05 (9)	C5—C6—C7—C8	−0.99 (15)
C3—N1—C2—O1	−178.32 (8)	C6—C7—C8—C9	1.21 (15)
C16—N1—C2—O1	−0.15 (14)	C7—C8—C9—C4	0.04 (14)
C3—N1—C2—C1	1.65 (9)	C5—C4—C9—C8	−1.51 (13)
C16—N1—C2—C1	179.81 (7)	C1—C4—C9—C8	−179.85 (8)
N2—C1—C2—O1	−179.29 (9)	N2—C1—C10—C11	−97.67 (9)
C4—C1—C2—O1	−60.86 (11)	C4—C1—C10—C11	136.02 (8)
C10—C1—C2—O1	64.25 (11)	C2—C1—C10—C11	13.23 (10)
N2—C1—C2—N1	0.74 (8)	N2—C1—C10—C15	78.35 (9)
C4—C1—C2—N1	119.18 (7)	C4—C1—C10—C15	−47.96 (9)
C10—C1—C2—N1	−115.71 (7)	C2—C1—C10—C15	−170.75 (7)
C1—N2—C3—O2	−174.97 (8)	C15—C10—C11—C12	−0.97 (13)
C1—N2—C3—N1	4.19 (9)	C1—C10—C11—C12	175.01 (8)
C2—N1—C3—O2	175.58 (8)	C10—C11—C12—C13	0.34 (14)
C16—N1—C3—O2	−2.58 (13)	C11—C12—C13—C14	0.48 (15)
C2—N1—C3—N2	−3.61 (9)	C12—C13—C14—C15	−0.65 (15)
C16—N1—C3—N2	178.23 (7)	C13—C14—C15—C10	0.00 (14)
N2—C1—C4—C5	10.83 (11)	C11—C10—C15—C14	0.81 (13)
C10—C1—C4—C5	135.55 (8)	C1—C10—C15—C14	−175.35 (8)
C2—C1—C4—C5	−99.79 (9)	C2—N1—C16—C17	72.64 (10)
N2—C1—C4—C9	−170.85 (7)	C3—N1—C16—C17	−109.43 (9)
C10—C1—C4—C9	−46.14 (10)	N1—C16—C17—C19	57.80 (10)
C2—C1—C4—C9	78.52 (9)	N1—C16—C17—C18	−179.18 (7)
C9—C4—C5—C6	1.73 (13)		

### Hydrogen-bond geometry (Å, º)

Cg1 is the centroid of the five-membered ring.

**Table:**

*D*—H···*A*	*D*—H	H···*A*	*D*···*A*	*D*—H···*A*
N2—H2···O2^i^	0.91 (1)	1.95 (1)	2.8512 (9)	174 (1)
C7—H7···*Cg*1^ii^	0.95	2.99	3.9308 (13)	170
C8—H8···O1^iii^	0.95	2.46	3.4069 (13)	172

Symmetry codes: (i) −*x*+1, −*y*+1, −*z*+1; (ii) *x*+1, *y*, *z*; (iii) −*x*+2, −*y*+1, −*z*+2.
